# Shifting Mountains of Electronic Waste

**DOI:** 10.1289/ehp.120-a148

**Published:** 2012-04-01

**Authors:** Naomi Lubick

**Affiliations:** Naomi Lubick is a freelance science writer based in Stockholm, Sweden, and Folsom, CA. She has written for *Environmental Science & Technology*, *Nature*, and *Earth*.

Local users are now the main source of electronic waste in Africa, but illegal imports of old computers, televisions, and other electronics devices from Europe, Asia, and North America still make their way there. That’s the finding of *Where Are WEEE in Africa?*, a new United Nations Environment Programme (UNEP) report about waste electronic and electrical equipment—also known as WEEE, or e-waste—in Benin, Côte d’Ivoire, Ghana, Liberia, and Nigeria.^1^ A large portion of these imports are of good quality, have a decent life expectancy, and bring many socioeconomic benefits, according to the report, but the rest is hazardous junk that is often resold and recycled under unsafe conditions.

Under the 1989 Basel Convention on the Control of Transboundary Movements of Hazardous Wastes and Their Disposal,^2^ e-waste that contains hazardous elements may not be exported to developing countries for disposal, although such waste can be sold as scrap inside a country. Nevertheless, at least 250,000 metric tons of e-waste still illegally enters the five African countries surveyed each year, comparable to about 5% of the e-waste produced in Europe.^1^ *Where Are WEEE?* coauthor Mathias Schluep of Empa, the Swiss Federal Laboratories for Materials Science and Technology, says most e-waste imports into West Africa come from Europe despite the presence of efficient European recycling facilities.

Local use of electronics equipment has jumped in each of the five countries, accounting for an estimated 50–85% of the e-waste reported in these countries in 2010.^1^ The report estimates that 30% of all secondhand imports don’t work, but that half the nonfunctioning items imported that year were repaired and resold locally.

E-waste often ends up in informal recycling centers, where it is sorted for reuse or broken down by hand and picked clean for valuable metals, then destroyed in inefficient, toxicant-producing settings, Schluep says. Open fires are tended by children, who are paid by dealers collecting metals such as copper. Schluep says girls who sell water to the workers in these settings also are exposed to the potentially toxic by-products released from the low-temperature fires.

The release of dioxins is on the rise from the burning of brominated flame retardants in plastics that house these components—dioxin emissions from cable burning in the greater Accra region, for instance, are estimated to correspond to about 0.3% of total dioxin emissions in Europe.^3^ While that number may sound small, Schluep says Accra’s tiny proportion, when extrapolated to the whole continent, adds up to a substantial amount. Recent measurements in Accra show increasing levels of polybrominated diphenyl ether flame retardants in breastmilk associated with informal recycling of e-waste.^4^

Unscrupulous sellers can get around the Basel Convention, which targets nonsalvageable items, by labeling e-waste as goods to be resold or donated. With millions of containers passing through European ports, “it’s impossible for a port authority to check them all,” says Ruediger Kuehr, executive secretary of the Solving the e-Waste Problem (StEP) Initiative, a multistakeholder initiative that counts industry, academia, governments, and nonprofits as its members. Loopholes allow sellers to ship items classified for reuse “even though it’s simply junk,” he says, but in the countries in the UNEP report, thriving refurbishment and repair businesses are “making a living, and also want to be environmentally sound.”

**Figure:**
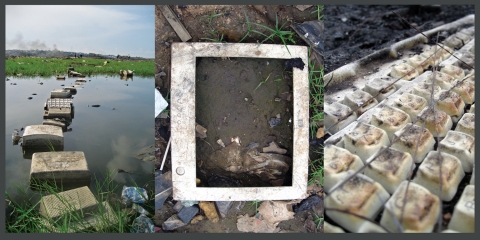
**Images from the Agbogbloshie scrap metal market and burning site in Accra, Ghana.** In the lefthand image, monitors are used as stepping stones across a creek. The waste heap in the background is a landfill dump, where workers are burning cables to recover copper. People burn all kinds of electronics at the informal recycling site to recover metals and other materials, releasing toxic by-products and losing a good deal of valuable material in the process. All images: Empa

Eric Williams, a professor at the Golisano Institute of Sustainability at the Rochester Institute of Technology in New York, is concerned that a disconnect between recyclers in Africa and the global market prevents them from selling back to Europe. “African recyclers could probably sell circuit boards to European metals refineries for more money than they would get by recycling the boards themselves,” he says. “Presumably the reason they don’t export circuit boards to Europe is that they’re not set up as an industry that can make long-term contracts and official export agreements. If this could be fixed, then the recycling of circuit boards, at least, wouldn’t be happening in Africa.”

Introducing recycling technology on a large scale would be difficult, Williams adds, because of infrastructure costs, maintenance, and a lack of other resources, including an educated workforce. Groups like Empa “can take the really bad things informal recyclers are doing and improve them,” he says, “but it will be hard to bring them up to good standards. There is no low-tech green and efficient solution to circuit board recycling.”

Kuehr supports a ban of e-waste shipments from developed to developing countries but applauds reuse of equipment through a second and third life as a way to reduce electronics’ large environmental footprint, which stems from resource-intensive manufacturing processes. He says a ban on e-waste intended for reuse could increase the market for even worse substitutes: brand-new but possibly low-quality equipment with a short lifetime, which brings along its own substantial environmental impacts.

Jim Puckett, cofounder of the nonprofit Basel Action Network, strongly disagrees on the value of trade bans and thinks African countries should establish legal barriers to accepting any e-waste. He also says manufacturers must take responsibility for electronics at the ends of their lives. Some manufacturers, such as Dell and Hewlett-Packard, are assisting in developing private programs to manage e-waste in Africa. “Manufacturers have to step up,” he says, and some “are starting to do so.”

Africa is not alone in its growth in domestic electronics users. Research in China and Peru has documented similar trends, with a burgeoning class of people who can afford to buy new and secondhand devices. By sometime between 2016 and 2018, domestic generation of e-waste in developing countries will outstrip generation in developed countries, says Williams.^5^ In the case of Peru, however, at least one company has profited by buying discarded electronics, often originating from the United States and elsewhere, and selling them to Aurubis,^6^ a state-of-the-art facility in Germany that is one of only five in the world equipped to properly process the hazardous components of circuit boards.
